# Finding Medical Photographs of Patients Online: Randomized, Cross-Sectional Study

**DOI:** 10.2196/55352

**Published:** 2024-06-24

**Authors:** Zack Marshall, Maushumi Bhattacharjee, Meng Wang, Abdul Cadri, Hannah James, Shabnam Asghari, Rene Peltekian, Veronica Benz, Vanessa Finley-Roy, Brynna Childs, Lauren Asaad, Michelle Swab, Vivian Welch, Fern Brunger, Chris Kaposy

**Affiliations:** 1 Department of Community Health Sciences Cumming School of Medicine University of Calgary Calgary, AB Canada; 2 School of Social Work McGill University Montreal, QC Canada; 3 Faculty of Law McGill University Montreal, QC Canada; 4 Department of Family Medicine Faculty of Medicine and Health Sciences McGill University Montreal, QC Canada; 5 Department of Anatomy & Cell Biology McGill University Montreal, QC Canada; 6 Faculty of Medicine Memorial University of Newfoundland St John's, NL Canada; 7 Renison University College University of Waterloo Waterloo, ON Canada; 8 Faculty of Medicine Universite de Montreal Montreal, QC Canada; 9 CKUT 90.3FM Radio McGill Montreal, QC Canada; 10 Health Sciences Library Memorial University St John's, NL Canada; 11 Bruyere Research Institute Ottawa, ON Canada; 12 Division of Population Health and Applied Health Sciences Faculty of Medicine Memorial University St John's, NL Canada

**Keywords:** patient photographs, privacy, informed consent, publication ethics, case reports

## Abstract

**Background:**

Photographs from medical case reports published in academic journals have previously been found in online image search results. This means that patient photographs circulate beyond the original journal website and can be freely accessed online. While this raises ethical and legal concerns, no systematic study has documented how often this occurs.

**Objective:**

The aim of this cross-sectional study was to provide systematic evidence that patient photographs from case reports published in medical journals appear in Google Images search results. Research questions included the following: (1) what percentage of patient medical photographs published in case reports were found in Google Images search results? (2) what was the relationship between open access publication status and image availability? and (3) did the odds of finding patient photographs on third-party websites differ between searches conducted in 2020 and 2022?

**Methods:**

The main outcome measure assessed whether at least 1 photograph from each case report was found on Google Images when using a structured search. Secondary outcome variables included the image source and the availability of images on third-party websites over time. The characteristics of medical images were described using summary statistics. The association between the source of full-text availability and image availability on Google Images was tested using logistic regressions. Finally, we examined the trend of finding patient photographs using generalized estimating equations.

**Results:**

From a random sample of 585 case reports indexed in PubMed, 186 contained patient photographs, for a total of 598 distinct images. For 142 (76.3%) out of 186 case reports, at least 1 photograph was found in Google Images search results. A total of 18.3% (110/598) of photographs included eye, face, or full body, including 10.9% (65/598) that could potentially identify the patient. The odds of finding an image from the case report online were higher if the full-text paper was available on ResearchGate (odds ratio [OR] 9.16, 95% CI 2.71-31.02), PubMed Central (OR 7.90, 95% CI 2.33-26.77), or Google Scholar (OR 6.07, 95% CI 2.77-13.29) than if the full-text was available solely through an open access journal (OR 5.33, 95% CI 2.31-12.28). However, all factors contributed to an increased risk of locating patient images online. Compared with the search in 2020, patient photographs were less likely to be found on third-party websites based on the 2022 search results (OR 0.61, 95% Cl 0.43-0.87).

**Conclusions:**

A high proportion of medical photographs from case reports was found on Google Images, raising ethical concerns with policy and practice implications. Journal publishers and corporations such as Google are best positioned to develop an effective remedy. Until then, it is crucial that patients are adequately informed about the potential risks and benefits of providing consent for clinicians to publish their images in medical journals.

## Introduction

Case reports are an important tool for medical, scientific, and educational purposes [1]. Written by practicing clinicians, peer-reviewed case reports provide relevant and timely medical information that contributes to evidence-based practice [1,2]. A large number of case reports are published each year; for example, 74,270 case reports were published in 2022 and indexed in PubMed.

Case reports often include images, including patient photographs [3,4]. Guidelines related to the publication of medical photographs in case reports often refer to overarching statements such as the Declaration of Helsinki, or slightly more specific policies such as the guidelines outlined by the Committee on Publication Ethics (COPE), or the Case Report (CARE) guidelines for case reports [5-7]. The Declaration of Helsinki states that research participants must be fully informed of any potential risks and benefits associated with the relevant study [8]. COPE guidelines provide clear recommendations for publishers, editors, and various research institutes on the topic of publication ethics [9]. Meanwhile, CARE guidelines are specific to case reports and seek to promote and improve their transparency, accuracy, and usefulness [10]. The CARE guidelines include a checklist with items such as deidentified patient information, informed consent, and patient perspective on the treatment they received [10]. While multiple guidelines exist, adherence is not mandatory; 1 study found that out of 50 journals, 76% did not adhere to any guidelines for publication of personal information [11]. Another study investigating CARE guideline adherence in 36 Indian medical journals found that only a third exhibited average adherence and that overall there was poor reporting of subject-informed consent [7].

With the growth of online publishing and advancements in technology, case reports from academic journals are widely available as web-based publications, and their reach has expanded to a larger audience [4]. While increased access to medical case reports may be beneficial, photographs from case reports published in academic journals are now also available in online image search results such as Google Images [3,4]. In such cases, patient photographs circulate beyond the original journal website and can be accessed by anyone using the internet. This raises ethical and legal concerns regarding patients’ informed consent and privacy of health information.

In the original study on this topic, drawing on a sample of case reports with patients who are transgender published between 2008 and 2015, at least 1 patient photograph was available on Google Images for 37% of the medical case reports in the sample [3]. Curious about whether the results would be the same for a random sample of medical case reports published more recently, the aim of this cross-sectional study was to provide systematic evidence that patient photographs from case reports published in medical journals appear in Google Images search results. Research questions for this study were (1) what percentage of patient medical photographs published in case reports are found in Google Images search results? (2) what is the relationship between open access publication status and image availability? and (3) do the odds of finding patient photographs on third-party websites differ between searches conducted in 2020 and 2022?

## Methods

### Study Design

The Strengthening the Reporting of Observational Studies in Epidemiology (STROBE) cross-sectional checklist was used when writing up results [12] (see Multimedia Appendix 1).

### Ethical Considerations

Research ethics approval was not required because the data were collected from case reports published in medical journals.

### Data Source and Study Population

PubMed includes a diverse range of medical journals and is “the most widely used database with biomedicine-related article abstracts” with over 36 million entries [13]. The efficient identification of a random sample was facilitated by the ways medical case reports are identified within PubMed. A structured search of PubMed was conducted to identify all indexed medical case reports published within a 1-year period between July 1, 2017, and June 30, 2018 (Search: “2017/07/01” [Date—Publication]: “2018/06/30” (Date—Publication) Filters: Case Reports). The search produced 23,589 results (the search was conducted on August 15, 2018).

### Sample Size

To determine sample size, a pilot study was conducted to inform an estimate of effect size and power. All medical case reports indexed in PubMed for the month of February 2018 were identified. This search produced 955 references. Full-text PDFs were retrieved for each reference and the documents were visually checked to see whether each case study included photographic images of patients. Of the 955 case reports published in English in February 2018 and indexed in PubMed, 370 (38.7%) included patient photographs. Based on the original study, it was anticipated that approximately 37% of the case reports with photographs would include at least 1 image found online. Using a 95% Cl and a 4% margin of error, a sample size of 585 was required.

### Data Collection and Measures

**Figure 1:**
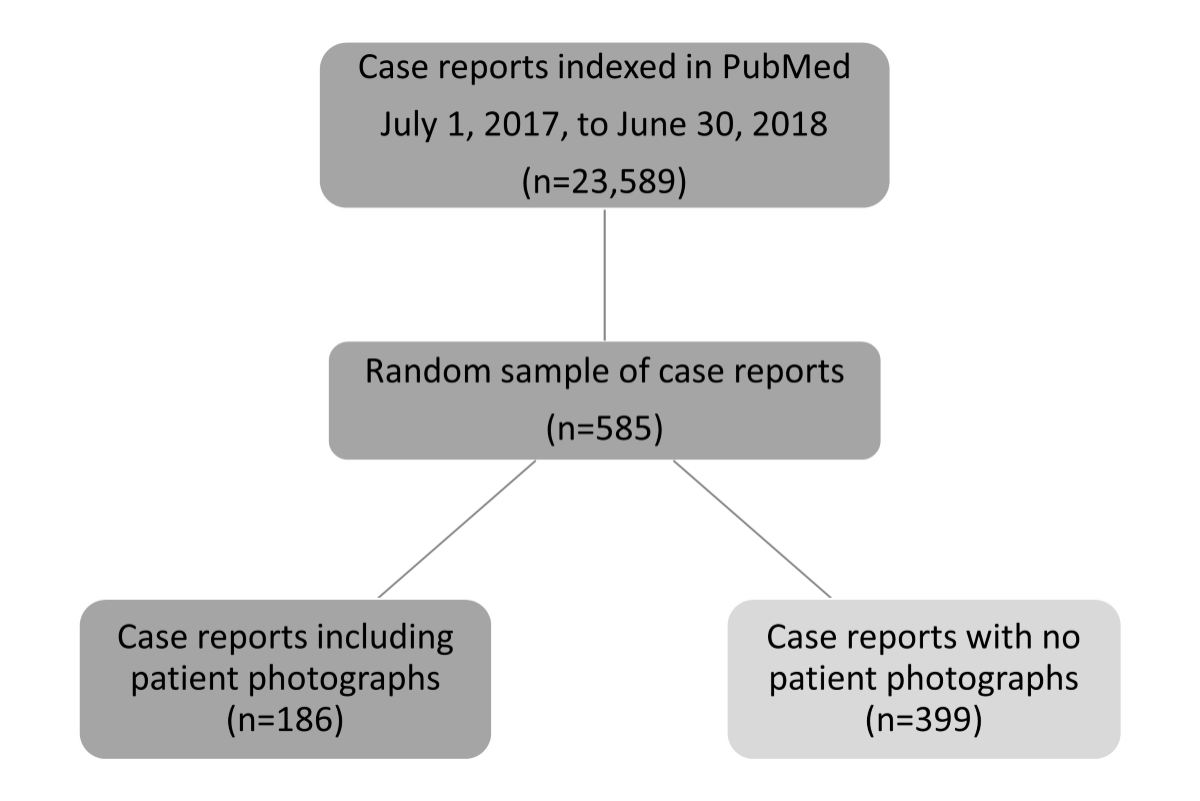
Identification of a random sample of case reports with patient photographs.

To identify the random sample for this study, the list of 23,589 case report references was exported from PubMed to Microsoft Excel (Microsoft Corp). A random number generator was used to assign a number to each of the references, and then the list of references was rank-ordered from the smallest to the largest random number. The first 585 references were selected in order, imported into EPPI-Reviewer (EPPI Centre) [14], and then full-text papers were uploaded for each reference (Figure 1).

For the 585 references, the full text of each case report was examined to determine whether the publication included clinical photographs of patients or not. The photographs from each publication were consecutively numbered on a hard copy, and then the information was entered into a Microsoft Excel spreadsheet, with a unique number for each case report and photograph. A total of 186 case reports included patient photographs, with a total of 598 patient photographs in the sample.

Two categories of data were collected—data at the case report level and data at the image level. At the image level, details were documented related to the specific part of the patient’s body that was photographed (eg, eye, face, or torso); the timing of the photograph (eg, pre- or posttreatment or during surgery); the gender and age of the patient as described in the body of the case report; whether the photograph was in color or not; and whether the authors had attempted to anonymize the photograph using image blurring or bars covering parts of the image. For each case report, 1 member of the team entered data about the images into the Excel spreadsheet. All data were then independently verified by a second team member.

At the case report level, data collection included author information, year of publication, open access status, and availability on Google Scholar, ResearchGate, and PubMed Central. To document the open access format for each of the case reports, 1 member of the team searched for each paper in the open database Unpaywall. The open access status of case reports is classified using colors [15]. The color classifications are (1) gold—published in an open access journal that is indexed by the Directory of Open Access Journals; (2) green—toll-access on the publisher page, but there is a free copy in an open access repository; (3) hybrid—free under an open license in a toll-access journal; (4) bronze—free to read on the publisher page, but without a clearly identifiable license; and (5) closed—all other papers, including those shared only on academic social networks or Sci-Hub. For the purposes of this analysis, open access included papers categorized as gold, green, hybrid, and bronze.

### Google Images Search

To determine whether it was possible to find photographs from the case reports on Google Images, searches were carried out for each of the 186 case reports that included patient photographs. Searches were conducted on a yearly basis from 2019 to 2022, using an approach referred to as algorithmic probing [16]. This analysis focuses on the results of the most recent searches conducted in 2022.

**Figure 2:**
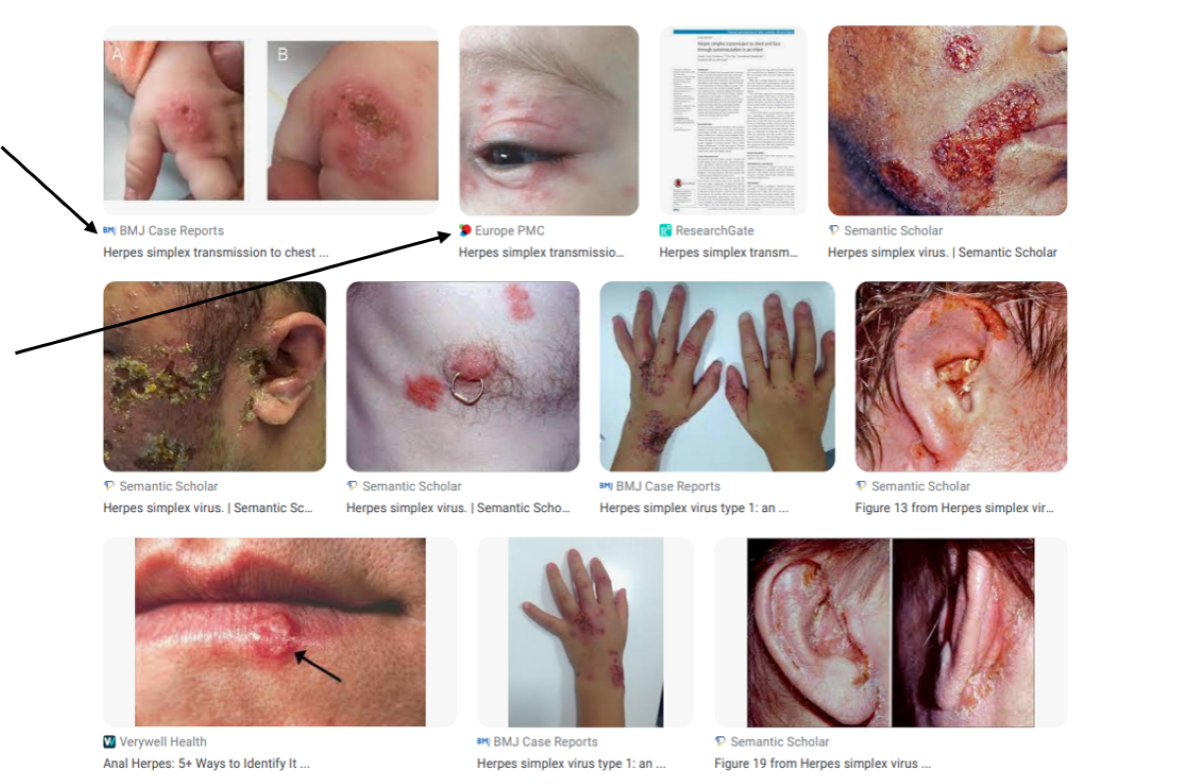
Sample of source links included in Google Images search results.

Three members of the research team (AC, HJ, and ZM) conducted manual searches on Google Images for each paper in the study sample using the same strategy first developed by Marshall et al [3]. For each reference, the researcher used the title of the case report in quotation marks as the text key. These searches were conducted using a Tor browser, “a proxy that masks the location information and browsing history of the user, allowing for anonymous use of the Internet” [17]. This browser was used to minimize the influence of Google’s personalization strategies to help prevent results from being skewed by historical searches conducted by team members [18]. Images of the search result pages were saved in PDF by date. One member of the research team then manually compared the search results in the PDF to the photographs in the published medical case report, circling the matching image using PDF editing software. Google Images search results also include a link directly under the image to the original source of the photograph. In Figure 2, for example, the first 3 images are from a case report [19] and include links to BMJ Case Reports, and Europe PMC. The link was extracted for each image and then each source was coded as a journal website, publisher website, research database (eg, Semantic Scholar), research repository (eg, ResearchGate), social media, professional association, or other. A second member of the team verified the results.

### Data Management

Data were entered into Microsoft Excel and screened for misentries (eg, spelling errors, empty cells, or shifted cells). The primary outcome variable was the availability of medical photographs on Google Images and was coded as “0” not found and “1” found. Secondary outcome variables included the image source and the availability of images on third-party websites over time. Missing data analyses were performed to screen the data for entry errors.

### Statistical Analysis

Sample characteristics were described using means, SDs, quantiles, and frequency distributions. The level of analysis is individual case reports rather than individual photographs. This is necessary for 2 reasons. First, some published figures contain more than 1 patient photograph. For example, a figure may include 4 images of a patient taken from different perspectives. Second, case reports included a range of 1 to 33 patient photographs. The relationship between multiple images found in 1 case report is different from multiple images found in separate case reports, and as a result, each photograph cannot be treated independently.

To better understand whether the characteristics of case reports (such as full-text availability on ResearchGate, PubMed Central, or Google Scholar, and open access status) were related to the availability of medical images on Google Images, chi-square tests and simple logistic regressions were conducted. To test if there is any trend for finding images on third-party websites over different searches over time, generalized estimating equations with a logit link and robust sandwich estimators were used. Odds ratios (OR) with 95% Cl were reported. *P*≤.05 was considered significant. Analyses were conducted using SAS (version 9.4; SAS Institute).

## Results

### Sample Demographics

From the sample of 585 case reports, 186 (31.7%) case reports had at least 1 patient clinical photograph. A total of 598 images were identified in these 186 medical case reports. Individual photographs were coded into five broad categories (1) the specific body part that was photographed; (2) patient sex as identified in the case report; (3) patient age (adult vs child); (4) the timing of the photograph (pretreatment, during surgery, autopsy, etc); and (5) whether the photograph was anonymized or not.

From the 186 case reports with 598 photographs, 309 (51.7%) were photographs of women, 278 (46.2%) were photographs of men, and 3 (0.5%) were photographs of trans women. Information about patient sex or gender was not provided for 8 (1.3%) of the photographs. Patients who were photographed ranged in age from 2 days to 93 years. A total of 412 (68.9%) photographs were taken of adult patients (older than 18 years), and 176 (29.4%) were photographs of infants, children, or teenagers younger than 18 years of age. Information about age was not provided for patients in 10 (1.7%) photographs.

Patient photographs most often included internal organs (eg, endoscopy, laparoscopy, or bronchoscopy; n=151 images, 25.3%). Other common types of photographs included limbs such as legs, arms, hands, or feet (n=109 images, 18.3%), or images of the abdomen or torso (n=53 images, 8.9%). A total of 110 (18.3%) out of 598 photographs included eye, face, and full-body photographs, including 65 (10.9%) that could potentially identify the patient. In terms of the context of when the photograph was taken, 403 (67.4%) were photographs of the patient’s condition pre- or posttreatment whereas 144 (24%) were photographs taken during surgery (Table 1).

**Table 1:** Patient demographic characteristics of medical images.

Patient demographic characteristics			Values, n (%)
**Gender**			
	Women	309 (51.7)	
	Men	278 (46.2)	
	Trans women	3 (0.5)	
	Unknown	8 (1.3)	
**Age (years)**			
	Adult (18 years or older)	412 (68.2)	
	Infant, child, or teenager (younger than 18 years)	176 (29.4)	
	Unknown	10 (1.7)	
**Type of photograph**			
	Internal organs or endoscopy	151 (25.3)	
	Limbs (legs, arms, feet, or hands)	109 (18.2)	
	Mouth	62 (10.4)	
	Torso or abdomen	53 (8.9)	
	Face	44 (7.4)	
	Eyes	41 (6.9)	
	Breasts or chest	36 (6.0)	
	Full body	25 (4.2)	
	Genitals	22 (3.7)	
	Ears	13 (2.2)	
	Head	13 (2.2)	
	Nose	2 (0.3)	
**Context of photograph**			
	Photograph of condition	403 (67.4)	
	Presurgery	7 (1.2)	
	During surgery	144 (24.1)	
	Specimen	39 (6.5)	
	Autopsy	3 (0.5)	
	Other	4 (0.7)	

### Open Access Status of Case Reports With Medical Images

Of the 186 case reports, 102 (54.8%) were closed access; among the closed-access reports, 66 (65%) case reports had at least 1 image found on Google Images. Of the 83 case reports that were open access, 76 (92%) had at least 1 image found on Google Images. From crude comparisons (*P*<.001), it appears that case reports with open access were more likely to have medical images visible as Google Images.

### Image Availability

**Figure 3:**
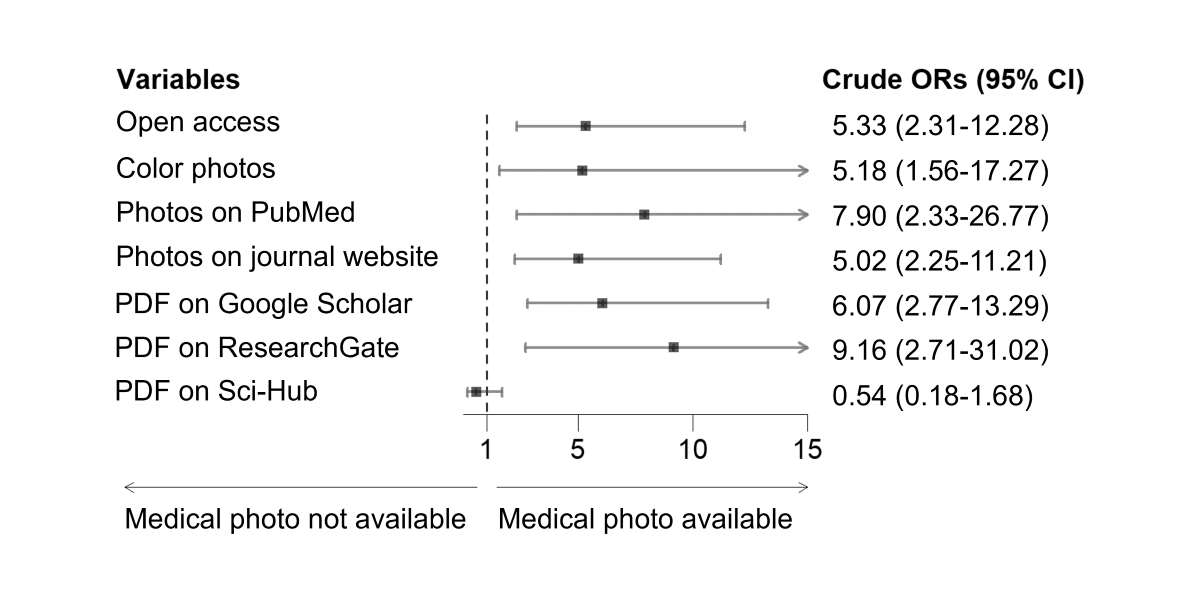
The relationship between the characteristics of case reports and availability of medical photographs by unadjusted ORs (simple logistic regression). OR: odds ratio.

For 76.3% (142/186) of the case reports, at least 1 image was found on Google Images. The odds were higher of finding an image from the case report online if the full-text paper was available on ResearchGate (OR 9.16, 95% Cl 2.71-31.02), PubMed Central (OR 7.90, 95% Cl 2.33-26.77), or Google Scholar (OR 6.07, 95% Cl 2.77-13.29) than if full-text was available solely through an open access journal (OR 5.33, 95% Cl 2.31-12.28), but all factors contribute to increased odds of locating patient images online (Figure 3).

### Image Source

To better understand where Google Images is obtaining patient photographs, information about data sources was extracted from the hyperlink under each of the images that were found online. Raw image sources included the journal website, publisher website, research database (eg, Semantic Scholar), research repository (eg, ResearchGate), social media, and professional associations. These were grouped into 2 main categories—journal websites or other websites (any third-party sources). A total of 51.0% of photographs came from the journal website, and 49.0% were from a third-party site. In 2021, 51.1% were from journal websites, and 48.9% from third-party sites. In 2022, the number of images from journal websites increased to 63.4%, while the number from third-party sites was 36.6%.

### Trend Over Time

Based on generalized estimating equations, after adjusting for individual study differences, compared with the search in 2020, patient photographs were less likely to be found on third-party websites based on the 2022 search results. Specifically, the odds of finding a patient photograph on a third-party site in 2022 were about 40% less likely, compared with the search done in 2020. This finding was statistically significant with OR 0.61, 95% Cl 0.43-0.87. The likelihood of finding a patient photograph on a third-party website was not significantly different between the search in 2021 and the search in 2020 (Table 2).

**Table 2:** Google Images findings over time based on generalized estimating equations.

Search	Odd ratio (95% CI)	*P* value
2021 vs 2020	1.04 (0.78-1.40)	.77
2022 vs 2020	0.61 (0.43-0.87)	.006

## Discussion

### Principal Results

The aims of this study were to identify what percentage of patient photographs published in medical case reports were found in Google Images search results, to better understand the relationship between open access publication status and image availability, and to verify whether there is a trend over time for finding patient photographs on third-party websites. Out of the 186 case reports that included clinical photographs, at least 1 photograph from the case report was available on Google Images for 142 (76.3%) references. The odds of finding an image from the case report online were higher if the full-text paper was available on ResearchGate (OR 9.16, 95% CI 2.71-31.02), PubMed Central (OR 7.90, 95% CI 2.33-26.77), or Google Scholar (OR 6.07, 95% CI 2.77-13.29) than if full-text was available solely through an open access journal (OR 5.33, 95% CI 2.31-12.28), but all factors contributed to an increased risk of locating patient images online. This study is the first of its kind to search Google Images for medical photographs from a random sample of case reports; as such there are no studies with which to compare results.

Findings from this study are notably higher than the results from earlier research, where 34 (37%) out of 94 case reports had at least 1 photograph accessible on Google Images [3]. While the difference in sample population may partially account for the disparity in outcomes, this study identified several additional variables that influenced the availability or unavailability of patient photographs on Google Images. For instance, finding images from the case reports online was more likely if the full-text paper was also available on ResearchGate, PubMed Central, or Google Scholar, compared to case reports solely accessible through open access publications.

To better understand how Google retrieves the images, the image source was recorded for all photographs found on Google Images and these results were compared over a 3-year time period. From 2020 to 2022, there was a notable change in where images were sourced, with a significant decrease in photographs housed on third-party websites such as ResearchGate and Semantic Scholar. This change may be linked to a recent legal judgment where Google was held liable for copyright infringement for displaying content with links to a third-party infringer’s website which was not the original publisher and owner of the copyrighted content [20].

### Limitations

The systematic, documented approach to searching for patient medical photographs on Google Images is a strength of this study. The primary challenge is that Google Images search results are not stable. Although the team attempted to manage as many factors as possible, including using the Tor browser to control for the influence of team member search histories, search results changed. Investigating the same data set yearly for over 3 years, sometimes the photographs were never found, while others were consistently located. The primary findings in this paper are based on the most recent searches in 2022, as the purpose of this study was not to demonstrate the ways search results change over time, but whether the images were found or not. Search results from 2020, 2021, and 2022 are available on request.

A further limitation is that the team did not investigate other image search engines or social media platforms where patient photographs might also appear. While the team was able to provide clear evidence using Google Images it would be an interesting avenue for future research to explore some alternate image search engines and platforms. In addition, the use of the Tor browser to minimize personalization in search results may not completely replicate the typical user experience and may have introduced a form of selection bias.

### Conclusions

From a clinical standpoint, the availability of patient photographs on Google Images presents both advantages and risks. Results demonstrated a high proportion of medical photographs from case reports on Google Images. While this concentration allows for wider accessibility and educational benefits, the public availability of these sensitive images online also raises ethical concerns with respect to the privacy of personal health information. Patients should be adequately informed about the possible impacts of providing consent for clinicians to publish their images in medical journals. Even if clinicians seek consent for their publication in case reports, it is not clear whether patients are informed about the possibility of photographs becoming available on Google Images and reaching unintended audiences, including the media and the general public. Similarly, it is not known whether clinicians themselves are aware of these risks. As such, they may not be in a position to ensure informed consent from their patients regarding the potential availability of their clinical images online. A recent content analysis of journal consent forms for the publication of patient photographs found that 55.5% (10/18) of consent forms related to 132 journals mentioned photographs being available to an audience outside of the journal website, but only 16.7% (3/18) addressed the possibility of the patient’s images being linked to journal or publisher social media platforms [21].

A lack of standardized guidelines poses a challenge to obtaining patient consent for publishing case reports with photographs. In addition to the policy and practice recommendations highlighted in earlier research, current findings underline the need for increased dialogue among academics, patients, governments, and industry. Discussions should focus on improving the consent process and establishing consistent practices and policies for publishing case reports with patient photographs. Study findings indicate that patient photographs are accessible on Google Images, even when published in closed-access case reports. Engagement with Google and other major online image repositories is critical to raise awareness of this issue and to seek input regarding the underlying causes and potential solutions. New policies should be implemented to ensure that patients are protected and that all stakeholders are aware of the risks involved in submitting clinical photographs to online medical journals. Accordingly, the next phase of this study focuses on qualitative interviews with case report authors, journal editors, publishers, and patients. The goal is to identify potential solutions to this complex ethical challenge, including responsive policies that will influence practices across academic publishing to maintain patient privacy.

## Appendix

Multimedia Appendix 1 Strengthening the Reporting of Observational Studies in Epidemiology (STROBE) cross-sectional checklist.

## Abbreviations

CARE: Case Report
COPE: Committee on Publication Ethics
OR: odds ratio
STROBE: Strengthening the Reporting of Observational Studies in Epidemiology

## Data Availability

The data sets generated and analyzed during this study are available from the corresponding author on reasonable request.
